# Supplementary material for: Parallel synapses with transmission nonlinearities enhance neuronal classification capacity
Source: PLoS Comput Biol. 2025 May 9;21(5):e1012285. doi: 10.1371/journal.pcbi.1012285 (PMC12063901; doi:10.1371/journal.pcbi.1012285)
Supplement: S1 Text — Supplementary materials including Figures and Tables (PDF) [file pcbi.1012285.s001.pdf]

S1 Text – Supplementary Materials for "Parallel Synapses with Transmission Nonlinearities Enhance Neuronal Classification Capacity"

Yuru Song and Marcus K. Benna (UC San Diego)

Numerical evaluation of the capacity of the sign-constrained Perceptron

The memory capacity of the sign-constrained Perceptron is a well-studied problem [1–4]. For clarity, we briefly describe the numerical simulations used to determine its capacity for finite N, which serves as the comparison benchmark throughout our work. The input patterns and their labels are sampled using the same process that generates the training data for our nonlinear parallel synapse models. The update rule follows the learning rule in [1].

To evaluate the algorithmic capacity of the sign-constrained Perceptron with this learning rule and with N input dimensions, we train the Perceptron to classify datasets of P random patterns. Successful classification requires correctly classifying all patterns in a given dataset. For each combination of N and P, we sample 10 different datasets. The value of P is gradually increased for each N to identify the critical capacity, P^*/N, at which the success rate drops to 0.5. The success rates for different N and P values are shown in Fig Ee and Ef in S1 Text.

Capacity of the restricted model with input sampled from a Gaussian distribution

The memory capacity of the restricted model was initially calculated using input values sampled from a uniform distribution, $\mathcal{U}(0, 1)$, as shown in Fig 2. To generalize this analysis, we also evaluate the memory capacity using input patterns drawn from a Gaussian distribution, $\mathcal{N}(0, 1)$. The procedure for determining memory capacity remains the same as described in Fig 2a, 2b and 2d, except for the change in input distribution. Our analysis focuses on the case with 5 parallel synapses per connection ($M = 5$). As shown in Fig Aa in S1 Text, the capacity scaling curve for Gaussian-distributed inputs is similar to that observed for the uniform distribution. The success rates for different N and P values are shown in Fig Eg in S1 Text.

Examples of the learned aggregate synaptic functions are presented in Fig Ab in S1 Text. Similar to the case with uniform input (Fig 2c), the aggregate synaptic functions also tend to be flatter near the center of the input range. The learned synaptic thresholds (Fig Ad in S1 Text) are correspondingly concentrated near the edges of the input range, but less so than for the uniformly distributed input case. The distribution of

the learned synaptic amplitudes (Fig Ac in S1 Text), and the relationship between a synapse's amplitude and its threshold also qualitatively mirror the uniform case (Fig Ae in S1 Text). However, the curve in Fig Ae in S1 Text is less sharp near the edges compared to Fig 2g. This difference is likely due to the shape of the input distribution: whereas the uniform distribution has sharp boundaries that lead to synaptic responses that strongly discriminate between inputs located close to the edges, the Gaussian distribution has tails from which few inputs are sampled, resulting in synaptic responses that are less sharply tuned to any particular boundary. In summary, the results for the restricted model remain qualitatively consistent when the input distribution is changed from uniform to Gaussian.

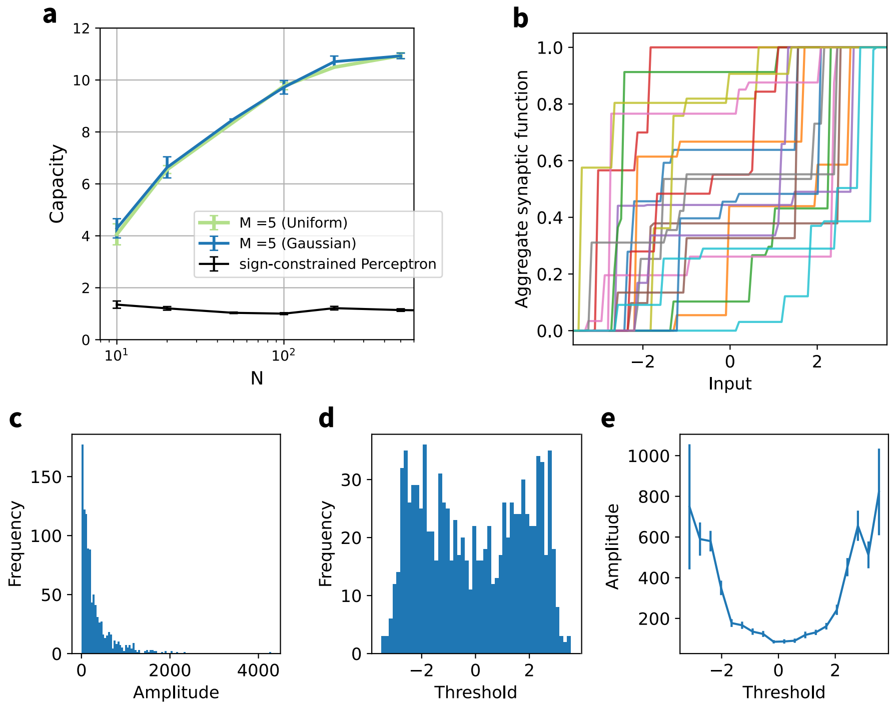

Figure A in S1 Text: Restricted model trained with input values sampled from a Gaussian distribution $\mathcal{N}(0, 1)$. **(a)**. The capacity P^*/N of a neuron with varying numbers of input axons and $M = 5$ parallel synapses per connection. As N increases, the capacity (blue) continues to grow similarly to the case with a uniform input distribution (green). **(b)**. Examples of the learned aggregate synaptic transmission functions, normalized by their maximum value, for $N = 200$, $M = 5$, and $P = 2200$. **(c)**. Histogram of synaptic amplitude values corresponding to the synapses in (b). **(d)**. Histogram of synaptic threshold values for the same synapses in (b). **(e)**. Relationship between synaptic amplitudes and their corresponding threshold values for the synapses in (b). The thresholds are binned as in (d), and the average amplitude of the respective parallel synapses in each bin is plotted.

Adding noise to the input patterns in the restricted model

In this section, we evaluate the memory capacity of the restricted model when noise is added to the input patterns. Specifically, each element of an input pattern, $\mathbf{x}_\mu$, is drawn i.i.d. from a uniform distribution,

$\mathcal{U}(0,1)$. Uniform noise, $\mathbf{u}_\mu$, is then added to each pattern, where each dimension of $\mathbf{u}_\mu$ is also drawn i.i.d. from a uniform distribution, $\mathcal{U}(0,\sigma)$. The noise level is controlled by σ. The classification label associated with $\mathbf{x}_\mu + \mathbf{u}_\mu$ remains the same as that of the original pattern, y_μ. To generate a noisy dataset, we repeat the process of adding different noise vectors Q times for each original pattern. Thus, for a dataset with P patterns, we obtain PQ noisy patterns.

We trained the restricted model on these noisy datasets and evaluated its memory capacity. The criterion for successful classification is strict: the model must correctly classify all PQ noisy patterns in the dataset to achieve success at a given P value. We varied Q from 20 to 50, with a fixed noise size of $\sigma = 0.01$ or 0.03. We focus on the case with $M = 5$ parallel synapses per connection. In Fig Ba in S1 Text, the memory capacity of the model trained on noisy data is shown. Compared to the model trained without noise (Fig 2), the capacity is lower. However, this is primarily due to the strict definition of successful classification we use here: for a given P, all PQ noisy patterns must be correctly classified. Even though it is difficult to achieve this for every single pattern, the values of the average classification accuracies for the PQ patterns are still close to one (Fig Bc in S1 Text) around the critical capacity. We also evaluated the model's classification accuracy on unseen noisy patterns at values of P/N close to the critical capacity. While the accuracy on unseen noisy data was slightly lower than on the noisy training data, it remained high (Fig Bc in S1 Text).

We also evaluated the classification capacity of the trained model on the original (unseen) patterns without noise (from which the training patterns were derived by adding noise). The model was trained solely on noisy training patterns and never encountered the original patterns during training. The capacity curve for this scenario is shown in Fig Bb in S1 Text. Again, this capacity is lower than that of the original model trained without noise, but significantly higher than that of the sign-constrained Perceptron. The memory capacity is higher when the training data has a lower level of noise. The number of noisy repeats Q in the dataset also influences the capacity curve (Fig Ba and Bb in S1 Text). For the classification capacity on the training data, larger Q generally leads to a slightly smaller capacity (Fig Ba in S1 Text). This is expected, as a higher Q introduces more noisy patterns, increasing the problem's difficulty under the strict criterion of correctly learning all PQ patterns. Conversely, for classification capacity on the original, unseen data, larger Q can lead to a slightly higher capacity (Fig Bb in S1 Text). This suggests that exposure to more noisy repeats improves the model's ability to generalize to the original patterns. Examples of the learned aggregate synaptic transmission functions of a trained model are shown in Fig Bg in S1 Text. The statistics of the trained parameters are summarized in Fig Bd, Be and Bf in S1 Text. Compared to the model of Fig 2, the model trained with noise has fewer synapses with large amplitudes and thresholds that are less concentrated near the edges of the input range (Fig Be and Bf in S1 Text).

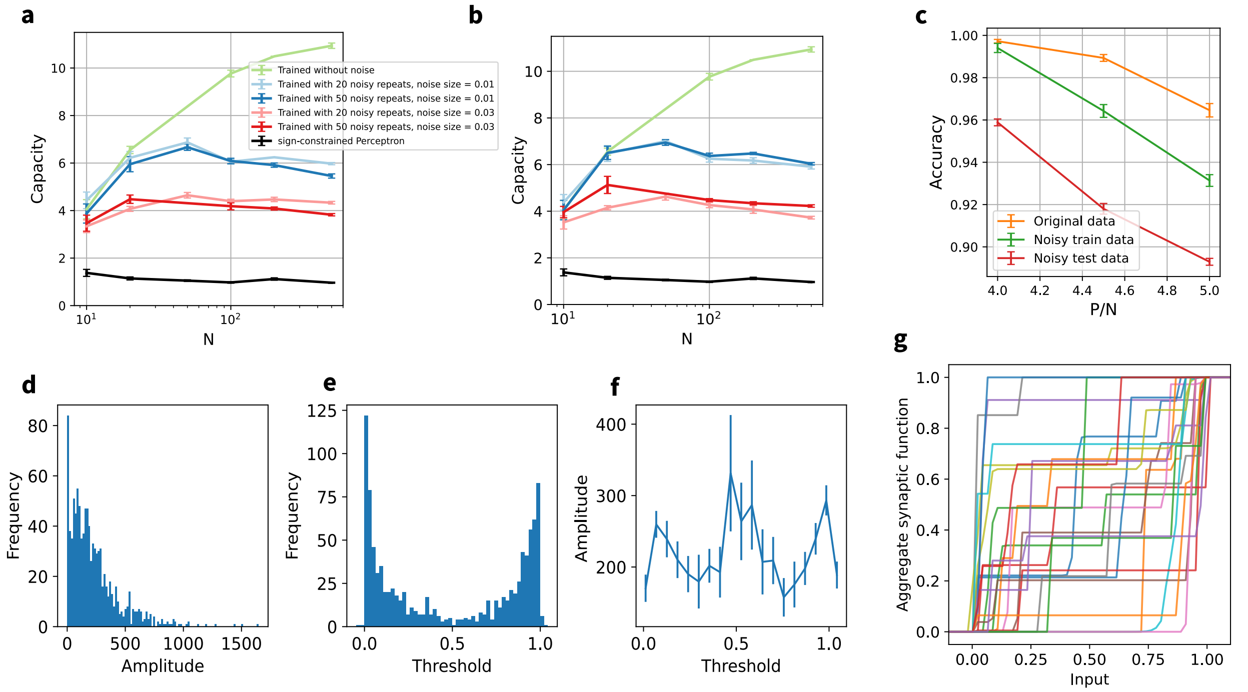

Figure B in S1 Text: Restricted model trained with noisy input. **(a)**. Memory capacity of the model trained on noisy data (blue and red curves) compared with the model trained on original, noise-free patterns (green curve) and the sign-constrained Perceptron (black curve). Noise is sampled from a uniform distribution with a standard deviation of either 0.01 (blue curves) or 0.03 (red curves). Each original pattern is augmented with either 20 (light blue and light red curves) or 50 (blue and red curves) noisy variations to form the training data. **(b)**. Memory capacity of the model trained on noisy data but evaluated on the original, unseen patterns. The training data consists of noisy patterns generated by adding noise to the original patterns, but the model has not been trained on the original patterns themselves. The legend is the same as in panel (a). **(c)**. Testing accuracy of the model on noisy, unseen data (red curve), original unseen patterns (orange curve), and noisy training patterns (green curve). Models are trained on noisy data with $Q = 50$ and a noise level of 0.03. Each model has $N = 100$ and $M = 5$. **(d)**. Histogram of amplitude values for all synapses in (g). **(e)**. Histogram of threshold values for all synapses in (g). **(f)**. The relationship between parallel synapses' amplitudes and their threshold values. The thresholds for all parallel synapses in (g) are divided into the same bins as in (e). We plot the average amplitude of the parallel synapses with thresholds in each bin. **(g)**. Examples of learned aggregate synaptic transmission functions, normalized by their maximum value, for $N = 200$, $M = 5$ and $P = 900$.

Effective synaptic weights and aggregate synaptic transmission functions

In this section, we examine the effective synaptic weights derived from the aggregate transmission functions. For the restricted model, each parallel synapse is parameterized using a sigmoid function, defined by its threshold, slope and amplitude (eqn. (1)). The amplitude $a^2_{i,j}$ determines the range of responses for each parallel synapse (Fig Ca in S1 Text), and the slope parametrizes the (maximum) derivative of the synaptic response (at the threshold). However, these parameters don't directly correspond to a practical definition of effective synaptic weights, because the aggregate synaptic transmission function in our model is nonlinear, and composed of the contributions of multiple parallel synapses.

Typically, synaptic weights are quantified by measuring the response of a synapse (or set of parallel synapses) to a presynaptic stimulus. In our case, the synaptic response varies with the stimulus magnitude in a nonlinear fashion due to the shape of the synaptic transmission functions. Nevertheless, we can define the effective synaptic weight as the ratio of the aggregate synaptic response to the magnitude of the input (Fig Cd in S1 Text). This definition (in combination with the nonlinearity of the synaptic response) implies that the effective weight of a set of parallel synapses depends on the size of the input, and we can construct a histogram of effective synaptic weights by sampling stimulus values from the input distribution. For the unrestricted model, the effective synaptic weights for each aggregate synaptic function can be derived using the same procedure. The histograms of amplitude parameters are shown in Fig Cb and Cc in S1 Text, and the histograms of effective synaptic weights are shown in Fig Ce and Cf in S1 Text. For the restricted model, the distributions of amplitudes (Fig Cb in S1 Text) and effective synaptic weights (Fig Ce in S1 Text) are qualitatively similar in shape. Both exhibit a unimodal distribution on a logarithmic scale (ignoring synapses with vanishingly small amplitudes). For the unrestricted model, the amplitude distribution (Fig Cc in S1 Text) exhibits a second peak at large values (around 1), likely due to the influence of the regularization term in the cost function (eqn. (6)). In contrast, the distribution of effective synaptic weights (Fig Cf in S1 Text) shows a heavy tail, with some weights taking on large values. These large effective synaptic weights result from the sharp initial increases in the aggregate synaptic functions (Fig 3b), due to the idealizations of modeling the synaptic responses with step functions in the unrestricted model and using a uniform input distribution with sharply delineated boundaries.

Capacity of the restricted model trained with synaptic pruning

In biological systems, synaptic wiring costs resources and energy [5]. To incorporate a wiring cost penalty into our simulations of the restricted model, we introduce synapse pruning. The synapse pruning process for a model in which each connection starts out with a given number M of parallel synapses is implemented as follows. During training, some synapses' amplitudes may decrease to zero, such that these synapses effectively become silent, with zero amplitude resulting in zero gradients also for the threshold and slope parameters (eqn. (5)). In our standard training process described above, silent synapses are reset by assigning them a new threshold randomly sampled from within the input range, and setting their amplitude to a small value. This allows the synapse to re-engage in subsequent updates and thus continue learning. However, as part of a pruning strategy to limit the total number of synapses, we can also choose to permanently prune silent synapses instead of resetting them. We consider different pruning scenarios based on the number of resets allowed before pruning occurs. One possibility is to prune a synapse as soon as it becomes silent for the first

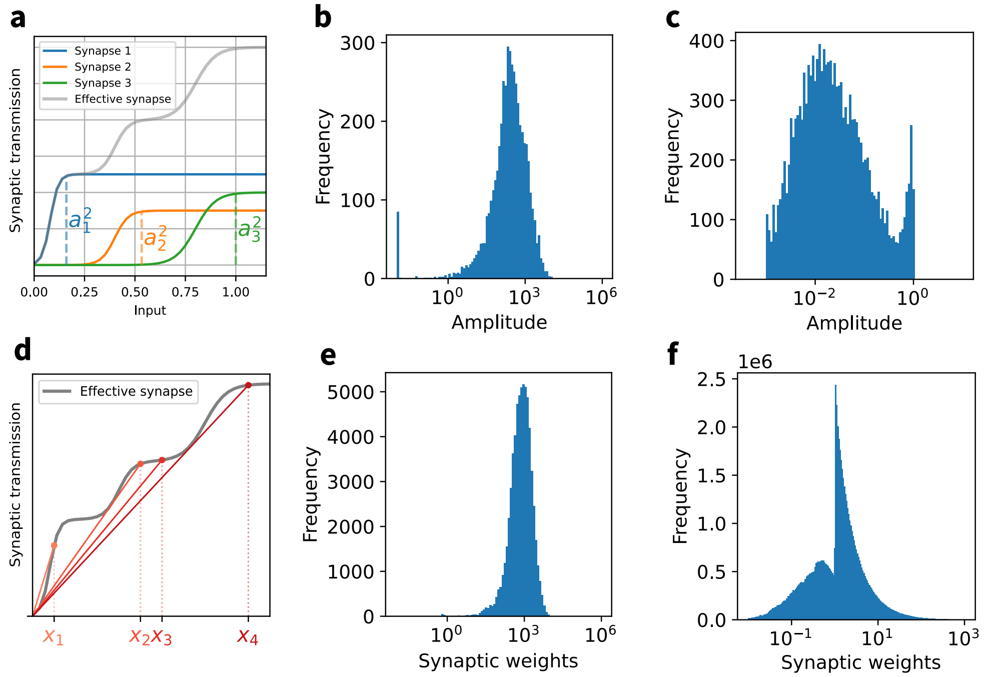

Figure C in S1 Text: Effective synaptic weights derived from aggregate transmission functions. **(a)**. Illustration of the amplitude parameter in the synaptic transmission function for the restricted model and the construction of the aggregate synaptic transmission function (effective synapse). **(d)**. Illustration of how effective synaptic weights are measured using inputs of different magnitudes. **(b) & (e)**. Amplitudes and effective synaptic weights for the restricted model with $N = 1000$ input dimensions and $M = 5$ parallel synapses per connection. The model is trained to successfully classify a dataset with $P = 11000$ data points, where patterns are sampled from a uniform distribution between 0 and 1. **(c) & (f)**. Amplitudes and effective synaptic weights for the unrestricted model with $N = 2000$ input dimensions. The model is trained to successfully classify a dataset with $P = 32000$ data points.

time. Other cases we simulated involve pruning a synapse after it has been reset 3, 10, 500 or 5000 times.

The resulting memory capacity curves for these pruning strategies are shown in Fig Da in S1 Text and the success rates for completely correct classification of P patterns are shown in Fig Em-p in S1 Text. Unsurprisingly, introducing synapse pruning leads to lower memory capacities compared to the standard training process, with stricter pruning criteria reducing memory capacity more. However, even with immediate pruning, the memory capacity remains higher than that of the sign-constrained Perceptron. Fig Db in S1 Text illustrates the average number of parallel synapses remaining for each neuronal connection under the different pruning strategies. In the standard training process, all neuronal connections retain the maximum number of parallel synapses ($M = 5$ in this case). When pruning is introduced, the number of parallel synapses per connection decreases, with stricter pruning leading to fewer connections that keep all (five of the) initially present parallel synapses. The average number of parallel synapses per connection is shown in Fig Dc in S1 Text, with stricter pruning criteria resulting in fewer parallel synapses per neuronal connection.

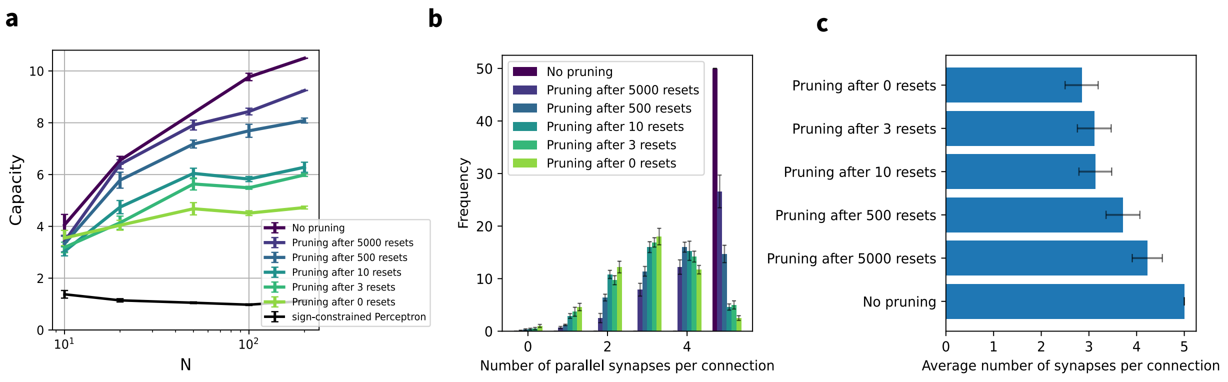

Figure D in S1 Text: Restricted model trained with synapse pruning. **(a)**. Memory capacity of the restricted model trained with synapse pruning. Results are shown for five different pruning strategies: immediate pruning (when the amplitude of a synapse becomes small), and pruning after 3, 10, 500 or 5000 resets. The dark purple curve represents the standard training process without synapse pruning. **(b)**. Average number of parallel synapses per connection for models trained with different levels of synapse pruning. Here we consider models with $N = 200$ and $M = 5$ for each pruning strategy. All models successfully classified their respective datasets. The error bars are standard errors of the mean, calculated from 10 different seeds. **(c)**. Average number of parallel synapses per connection for the same models as in (b). The error bars are standard errors of the mean, calculated from 10 different seeds.

Comparison of two types of neural networks for MNIST task

Here we enumerate the total number of parameters in networks with nonlinear parallel synapses and networks with standard linear synapses. We use D_{in} as the number of neurons in the input layer, which is $28 * 28$ for both types of network. D_{out} denotes the number of neurons in the output layer, which is 10 for both types of network. D_{hidden} is the number of neurons in the hidden layer. We choose D_{hidden} as $5, 10, 20$ and 30 for networks with parallel synapses. We set the number of parallel synapses per connection to 3, i.e., $M = 3$. For networks with nonlinear parallel synapse in the hidden-output connection, the total number of parameters is $(D_{\text{in}} + 1)D_{\text{hidden}} + (3MD_{\text{hidden}} + 1)D_{\text{out}}$, including the bias terms. For networks with only single linear synapses, the total number of parameters is $(D_{\text{in}} + 1)D_{\text{hidden}} + (D_{\text{hidden}} + 1)D_{\text{out}}$, also including the bias terms. Therefore, we opt for a slightly larger D_{hidden} for networks with linear synapses to achieve a fair comparison. The parameter counts for the two types of networks are shown below in Table A in S1 Text.

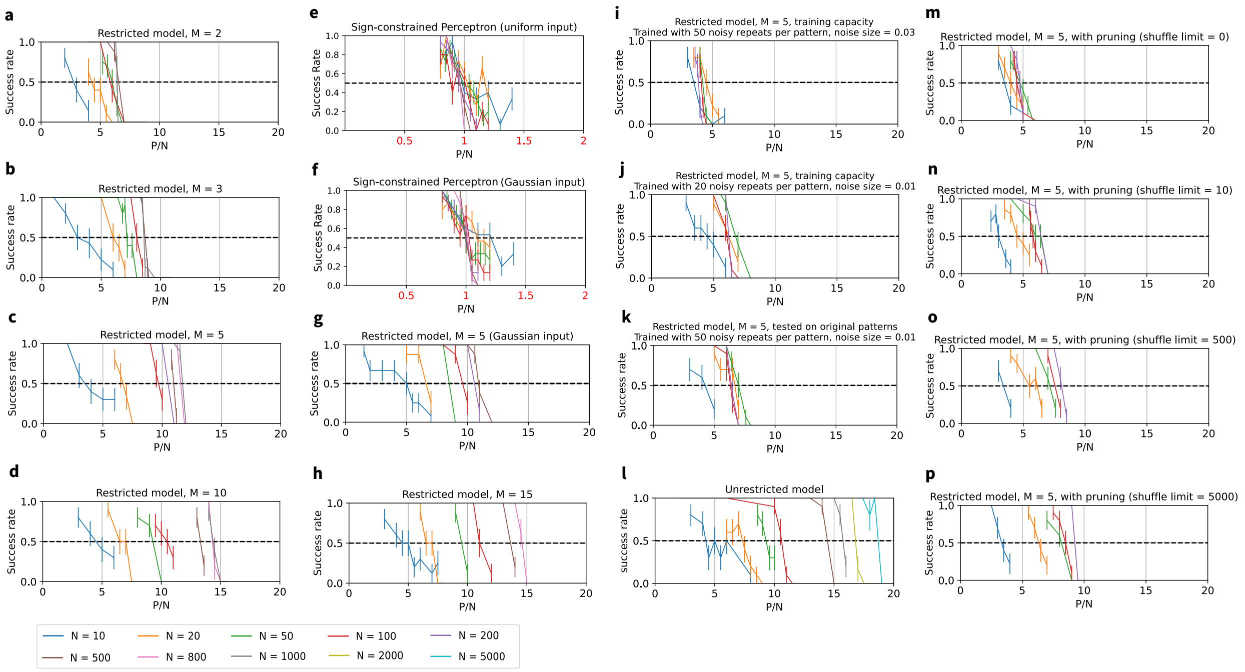

Figure E in S1 Text: Success rate curves for numerical simulations. **(a-d & h)**. Restricted model trained on random patterns sampled from a uniform distribution between 0 and 1. The number of parallel synapses per connection equals $M = 2$, 3, 5, 10 and 15, respectively, in these plots. **(e)**. Sign-constrained Perceptron trained on random patterns sampled from a uniform distribution between 0 and 1. (The x-axis is zoomed in between 0 and 2 for better visualization here and for the following panel, while other panels have x-axes ranging from 0 to 20). **(f)**. Sign-constrained Perceptron trained on random patterns sampled from a standard normal distribution. **(g)**. Restricted model with $M = 5$ parallel synapses per connection trained on input patterns sampled from a Gaussian distribution. **(i-k)**. Restricted model with $M = 5$ parallel synapses per connection trained on input patterns with noise. In (i) and (k), each of the P original patterns is turned into 50 different noisy patterns. In (j), each original pattern becomes 20 different noisy patterns. Panels (i) and (j) show success rates on the training patterns with noise, while (k) shows success rates on the original (unseen) patterns without noise. **(l)**. Unrestricted model trained on random patterns. **(m-p)**. Restricted model trained with synapse pruning. The criteria for synapse pruning from (m) to (p) vary as follows: (m) pruning immediately once a synapse becomes silent, (n) pruning after 10 resets, (o) pruning after 500 resets, (p) pruning after 5000 resets.

Network type	#parameters	Network type	#parameters
$D_{\text{hidden}} = 5,$ 3 parallel synapses	4370	$D_{\text{hidden}} = 6,$ linear synapses	4780
$D_{\text{hidden}} = 10,$ 3 parallel synapses	8760	$D_{\text{hidden}} = 11,$ linear synapses	8755
$D_{\text{hidden}} = 20,$ 3 parallel synapses	17510	$D_{\text{hidden}} = 22,$ linear synapses	17500
$D_{\text{hidden}} = 30,$ 3 parallel synapses	26260	$D_{\text{hidden}} = 33,$ linear synapses	26245

Table A in S1 Text: Comparison of parameter numbers in networks with parallel synapses (left two columns) and networks with single linear synapses (right two columns).

Below we record the accuracy of both types of networks on the testing set after 50 epochs of training in Table B in S1 Text. The standard deviation is calculated from 20 trained networks with different initialization

seeds.

Network type	Accuracy	Network type	Accuracy	Gain
$D_{\text{hidden}} = 5,\ 3$ parallel synapses	$91.42 \pm 0.06\%$	$D_{\text{hidden}} = 6$, linear synapses	$89.93 \pm 0.11\%$	**1.49%**
$D_{\text{hidden}} = 10,\ 3$ parallel synapses	$94.92 \pm 0.06\%$	$D_{\text{hidden}} = 11$, linear synapses	$92.93 \pm 0.04\%$	**2.00%**
$D_{\text{hidden}} = 20,\ 3$ parallel synapses	$96.41 \pm 0.05\%$	$D_{\text{hidden}} = 22$, linear synapses	$94.90 \pm 0.03\%$	**1.51%**
$D_{\text{hidden}} = 30,\ 3$ parallel synapses	$96.77 \pm 0.03\%$	$D_{\text{hidden}} = 33$, linear synapses	$96.02 \pm 0.02\%$	**0.75%**

Table B in S1 Text: Comparison of the classification accuracy in networks with parallel synapses (left two columns) and networks with single linear synapses (middle two columns). The accuracy improvements from using parallel synapses are shown in the right column.

Learned aggregate synaptic function in neural networks

For the network depicted in Fig 4a (with $D_{\text{hidden}} = 10,\ 3$ parallel synapses), we can also visualize the activation distributions of individual hidden units, as shown in Fig F in S1 Text. The input patterns are from the testing set of the MNIST dataset. For each hidden unit, we collect its activations (before the nonlinear synaptic transmission function) across various input patterns. These activations serve as the input values to the parallel synapses. Each hidden unit is connected to each output unit via a set of parallel synapses. A set of parallel synapses can be represented by their aggregate synaptic function. Thus, for each hidden unit, we can plot all the aggregate synaptic functions connecting this hidden unit to all the output units, which is also visualized in Fig F in S1 Text. In addition, Fig G in S1 Text shows the distributions of parameters (slope, amplitude and threshold) in the parallel synapses from the same network.

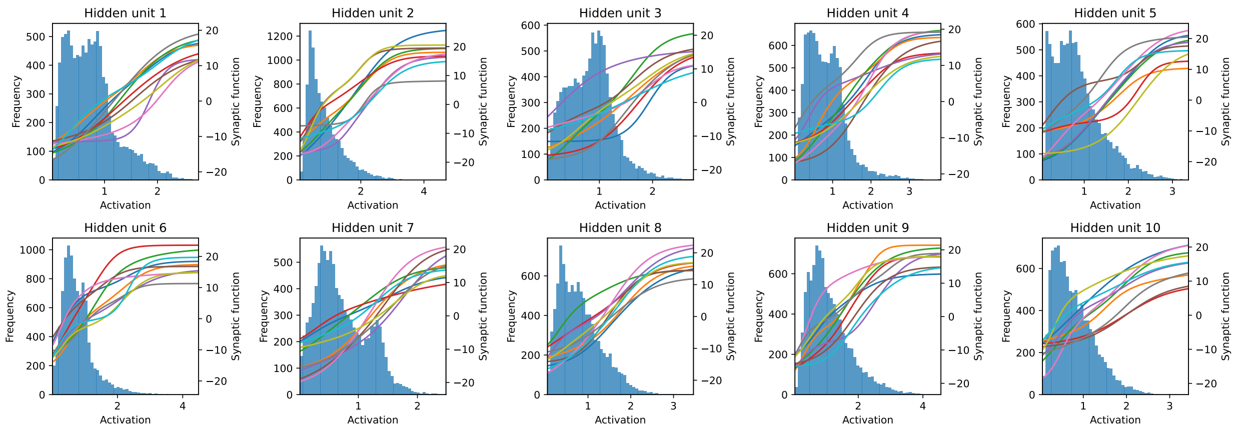

Figure F in S1 Text: Histograms of hidden unit activations in the two-layer neural network with parallel synapses, overlaid with learned aggregate synaptic transmission functions. The x-axis is the total activation of the corresponding hidden unit. The left y-axis is the frequency of activation values. The right y-axis is the value of the aggregate synaptic function. Each panel corresponds to one hidden unit. The histograms collect activation values from all patterns in the testing set of the MNIST dataset. The lines are aggregate synaptic functions connecting each hidden unit to all output units, with 10 output units in total.

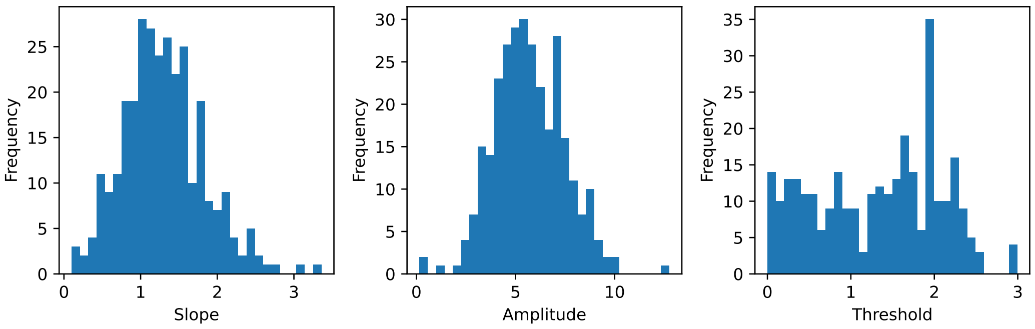

Figure G in S1 Text: Histograms of parameters in parallel synapses from the same network as in Fig 4, with slope (left), amplitude (middle) and threshold (right).

**Parameter type**	**Parameter value**
optimizer	Adam [6] with default PyTorch setting
batch size	256
learning rate for parallel synapses	0.02
learning rate for bias in batch normalization	0.01
learning rate for other parameters	0.001
learning rate scheduler	StepLR in PyTorch
step size of learning rate scheduler	5
decrease factor of learning rate scheduler	0.5
number of epochs	50
activation function of hidden layer	Softplus
weight initialization for linear synapses	Kaiming uniform distribution [7]
initialization for amplitude parameters	uniform distribution $\mathcal{U}(2, 3)$
initialization for slope parameters	uniform distribution $\mathcal{U}(1, 2)$
initialization for threshold parameters	uniform distribution $\mathcal{U}(0, 3)$

Table C in S1 Text: Hyperparameter settings for training neural networks.

References

[1] Amit DJ, Wong KYM, Campbell C. Perceptron learning with sign-constrained weights. Journal of Physics A: Mathematical and General. 1989;22(12):2039. doi:10.1088/0305-4470/22/12/009.

[2] Amit DJ, Campbell C, Wong KYM. The interaction space of neural networks with sign-constrained synapses. Journal of Physics A: Mathematical and General. 1989;22(21):4687. doi:10.1088/0305-4470/22/21/030.

[3] Nadal JP. On the storage capacity with sign-constrained synaptic couplings. Network: Computation in Neural Systems. 1990;1(4):463–466.

[4] Brunel N, Hakim V, Isope P, Nadal JP, Barbour B. Optimal information storage and the distribution of synaptic weights: perceptron versus Purkinje cell. Neuron. 2004;43(5):745–757.

[5] Bullmore E, Sporns O. The economy of brain network organization. Nature reviews neuroscience. 2012;13(5):336–349.

[6] Kingma DP, Ba J. Adam: A Method for Stochastic Optimization; 2017.

[7] He K, Zhang X, Ren S, Sun J. Delving deep into rectifiers: Surpassing human-level performance on imagenet classification. In: Proceedings of the IEEE international conference on computer vision; 2015. p. 1026–1034.
